# Electroacupuncture ameliorates peptic ulcer disease in association with gastroduodenal microbiota modulation in mice

**DOI:** 10.3389/fcimb.2022.935681

**Published:** 2022-08-19

**Authors:** Xiaoshuang Li, Feiyu He, Xuan Tuo, Yuanming Qiu, Jingjing Guo, Yiming Wu, Xianjun Meng, Zongbao Yang

**Affiliations:** ^1^ Department of Traditional Chinese Medicine, School of Medicine, Xiamen University, Xiamen, China; ^2^ Eye Institute, School of Medicine, Xiamen University, Xiamen, China

**Keywords:** peptic ulcer disease, electroacupuncture, gastric microbiota, duodenal microbiota, shousanli (LI10), zusanli (ST36), 16S rDNA sequencing

## Abstract

Peptic ulcer disease (PUD) is a common disease and frequently encountered in the clinic. Accumulating evidence suggests that PUD is associated with the gastrointestinal microbiota. Electroacupuncture (EA) is an improved version of acupuncture, which can improve the clinical effect by increasing the stimulation and delivering appropriate electrical pulses to needles. This method has been widely used in the treatment of peptic ulcer disease. However, its effect on gastrointestinal microbiota remains unclear. Therefore, in the present study, the ameliorative effect of EA was evaluated on the gastroduodenal mucosa, and the regulatory effect of the gastroduodenal microbiota was assessed in PUD mice. A total of 48 male Kun Ming mice were randomly divided into the following groups: normal control group (NC), PUD model group (PUD), Shousanli group (LI10), and Zusanli group (ST36) (n=12). The mice in groups LI10 and ST36 were treated with EA at LI10 and ST36, respectively. This intervention was continued for 7 days. Subsequently, we evaluated the morphological changes in the gastric and duodenal mucosa, and specific indices were measured, including the contents of serum dopamine (DA), the trefoil factor (TFF), and the vasoactive intestinal peptide (VIP). In addition, the gastric and duodenal microbiota were assessed *via* 16S ribosomal DNA sequencing. The results indicated that EA at LI10 or ST36 significantly reduced the injury of the gastroduodenal mucosa in PUD mice. The gastric microbial community structure of the groups LI10 and ST36 was similar to that of the NC group following comparison with the microbial community structure of the PUD model group. Moreover, the abundance of *Firmicutes* in the stomach was decreased, whereas that of *Bacteroidetes* was increased, and the abundance of *Firmicutes* in the duodenum was decreased. Furthermore, the microbial diversity and richness of the gastric microbiota in group LI10 were also significantly increased, and the serum dopamine and trefoil factor levels in group ST36 were significantly increased. Therefore, it is suggested that EA ameliorating PUD is in association with improving the levels of DA and TFF and regulating the relative abundances of *Firmicutes* and *Bacteroidetes* in the gastric microbiota.

## Introduction

Peptic ulcer disease (PUD) is a common gastrointestinal disease, which is characterized by gastric and duodenal mucosal erosion, bleeding, ulcer, etc. According to a systematic review from Azhari et al. (2018), the highest annual incidence of all PUD was 141.9 per 100,000 individuals each year. Patients with PUD presented with gastroduodenal hemorrhage, perforation, and obstruction, which are the main reasons for the high mortality of PUD (Dunlap and Patterson, 2019). PUD is considered to be caused by several factors, mainly including *Helicobacter pylori* infection and the use of non-steroidal anti-inflammatory drugs (NSAIDs) (Kavitt et al., 2019; Narayanan et al., 2018). Excessive drinking, smoking, inappropriate dietary factors, and lifestyle can also cause PUD (Yegen, 2018). The pathogeneses of this disease are diverse, and the current research has generally shown that the occurrence of PUD is related to the imbalance between the invasive factors and defensive factors of the gastrointestinal mucosa (Oluwole, 2015; Sierra et al., 2018). Research that has been conducted in precious years (Khosravi et al., 2014; Oh et al., 2016) has shown that the gastrointestinal microbial balance is involved in the occurrence and development of PUD. Decreased gastric and duodenal microbial diversity and dysbacteriosis are often observed in patients with PUD (Chen et al., 2018b). The clinical treatment approaches for PUD include the proton pump inhibitors or the eradication of *H. pylori*, which requires the use of antibiotic therapy (Kamada et al., 2021). However, an increased risk of enteric infections has been noted in patients receiving proton pump inhibitors (PPIs). The overuse of PPIs leads to a significant shift of the gastrointestinal microbiota towards a less healthy state (Minalyan et al., 2017).

Acupuncture is a complementary and alternative therapy in traditional Chinese medicine, which is widely used to treat various diseases, including PUD (Tian et al., 2017). Electroacupuncture (EA) is an improved version of acupuncture, which can improve the clinical effect by increasing stimulation and delivering appropriate electrical pulses to needles. However, the mechanism by which EA acts on PUD is unclear. Previous studies have shown that EA can regulate substance P, gastrointestinal peptides, gastric peristalsis, and gastric secretion; increase the level of prostaglandin E2 in the gastric mucosa; and enhance the protective effect of gastric mucosal cells (Yang et al., 2014) (Luo et al., 2014). Moreover, previous studies have also confirmed that EA can effectively restore the membrane metabolism and energy metabolism of PUD, promote the repair of gastric mucosal injury, and reverse the development of this disease (Zhang et al., 2018; Shen et al., 2019). However, the impact of EA treatment on the gastroduodenal microbiota has been rarely assessed in PUD. Therefore, it is of considerable importance to investigate the effects of EA on the gastrointestinal microbiota of subjects with PUD. This will be helpful to further reveal the mechanism of EA treatment in PUD.

In the present study, alcohol-induced PUD mice were treated with EA. The aim of the experiments was to explore the effects of this method on the gastroduodenal microbial community structure in mice with PUD using 16S ribosomal (r)DNA sequencing. Moreover, serum dopamine (DA), trefoil factor (TFF), and vasoactive intestinal peptide (VIP) were detected to further explore the possible mechanism by which EA can repair the gastroduodenal mucosa. This information will provide more theoretical basis for the clinical treatment of PUD.

## Materials and methods

### Animals

A total of 48 Kun Ming (KM) male mice, weighing 26–32 g, were obtained from Zhejiang Weitong Lihua Experimental Animal Technology Co., Ltd. All mice were housed at the specific pathogen-free (SPF)-certified animal facility in the Experimental Animal Center of Xiamen University. The room temperature was maintained at 22°C–26°C, and the humidity was maintained at 60%–70% under a 12-h light/dark cycle. All the mice were allowed water *ad libitum* throughout the study and were acclimated for 1 week prior to the experiments. The present study was approved by the Ethics Committee of the Laboratory Animal Center, Xiamen University (permit number XMULAC20190142). All the mice were randomly divided into the following groups: normal control group (NC), PUD model group (PUD), Shousanli group (LI10), and Zusanli group (ST36). Each group consisted of 12 mice. The disposal of animals in the whole experimental process followed the guiding opinions on treating experimental animals issued by the Ministry of Science and Technology of the People’s Republic of China.

### Alcohol-induced PUD murine model

All groups were fasted for 24 h before model construction. The murine model of PUD was prepared by oral gavage of absolute ethanol at the dose of 0.1 ml/10 g (Zhao et al., 2020). The mice in group PUD were sacrificed under anesthesia. The stomach and duodenum were dissected; then, the erosion and bleeding of the gastric mucosa and congestion and edema of the duodenum were observed with naked eyes. The PUD model was then successfully replicated in groups LI10 and ST36. The mice in the group NC were given corresponding volume of normal saline.

### EA treatment

**Figure 1:**
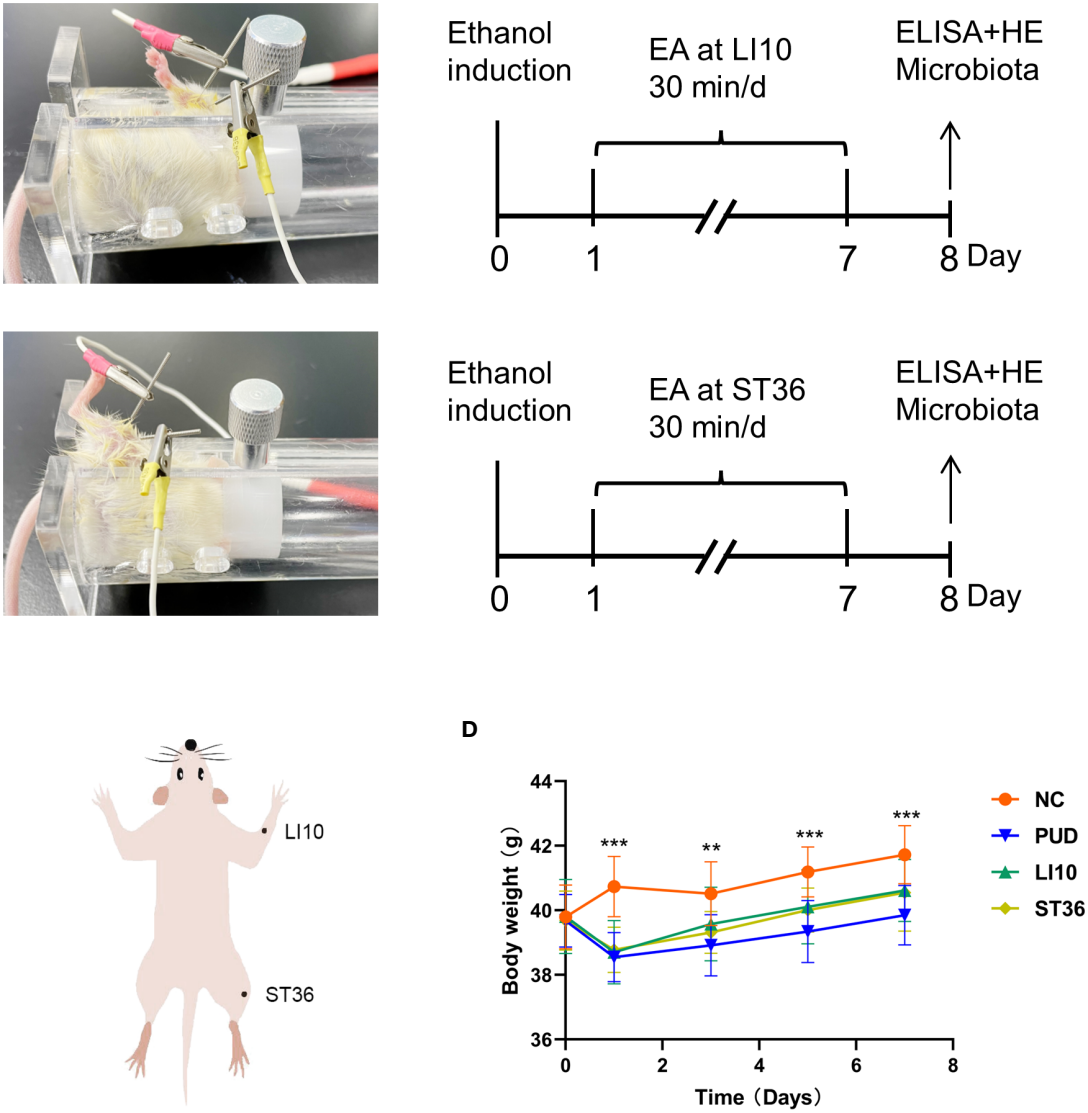
Experimental procedures and the changes in body weight of mice. **(A)** Experimental procedures. EA, electroacupuncture; LI10, Shousanli; ELISA, enzyme-linked immune sorbent assay; HE, hematoxylin and eosin staining. **(B)** Experimental procedures: ST36, Zusanli. **(C)** The position of Shousanli (LI10) and Zusanli (ST36). **(D)** Changes in body weight of mice in each group. *p< 0.05, **p< 0.01, ***p< 0.001.

One day following modeling, the mice of the groups NC and PUD were fixed with a mice holder for 30 min once daily for 7 days in the absence of additional intervention. The mice in groups LI10 and ST36 were treated using EA at LI10 and ST36, respectively. Subsequently, a needle (0.16×7 mm; Tianjin PengYi Medical Instrument Co., Ltd., China) was inserted into the acupoint, and an additional needle of the same size was inserted into the non-acupoint position 1 mm next to the acupoint. These two needles were connected with a G6805-I Electroacupuncture Stimulator (Qingdao XinSheng Industrial Co., Ltd., China). The output parameters were sparse and the dense waves (sparse wave, 4 Hz; dense wave, 50 Hz) and voltage (2–4 V). The intensity valve on the apparatus was adjusted to 1.0 mA, while EA was performed on each mouse for 30 min once daily for 7 days. The procedures of EA are shown in **Figures 1A, B**. The locations for these two acupoints were determined according to “The Veterinary Acupuncture of China” (**Figure 1C**). The acupoints were alternately performed at the left or right lower limbs, and the penetration depth was maintained at 3–4 mm.

### Sample collection

The mice’s body mass was monitored on the first, third, fifth, and seventh days during treatment. After EA, all mice were sacrificed following a 24-h fast for subsequent analysis. Six mice in each group were randomly selected for evaluation of ulcer index and histopathological examination; the rest were used for 16S rDNA testing.

### Evaluation of ulcer index

After the mice were sacrificed, the stomach was cut along the greater curvature and cleaned with 0.9% NaCl solution. The area of gastric ulcer was measured by vernier caliper, and the ulcer index was calculated. The degree of gastric mucosal injury was scored according to the standard of (Guth et al., 1979): 0, gastric mucosal integrity; 1, the gastric mucosal had a small spot erosion; 2, the ulceration length<2 mm; 3, the ulceration length was 2–3 mm; 4, the ulceration length was 3–4 mm; and 5, the ulceration length >4 mm. The score was multiplied by 2 when the width of ulceration was >1 mm. The degree of duodenal mucosal injury was evaluated according to the method of Moraes et al. (1978): 0, normal mucosa; 1, mucosal congestion and edema; 2, mucosal erosion and bleeding; 3, superficial ulcer; 4, deep ulcer; and 5, ulcer perforation.

### H&E staining of gastric and duodenum tissue

The gastric tissue (0.5 cm × 0.5 cm) and the duodenum tissue (0.5 cm) were collected and washed by sterile 0.9% NaCl solution on aseptic table. These tissues were placed in 4% paraformaldehyde solution and fixed for 48 h, gradient ethanol dehydration was performed, and paraffin was embedded. These tissues were sliced into 5-µm thick specimens and stained with hematoxylin and eosin (H&E). Observations were made under a light microscope to determine levels of pathological damage.

### The expressions of DA, TFF and VIP in mouse serum

Blood was taken from all mice before death into a centrifuge tube, coagulated for 30 min, centrifuged at 3,000 R for 20 min, and the serum was stored in a −80°C refrigerator for analysis. Enzyme linked immune sorbent assay (ELISA) was used to measure the levels of DA, TFF, and VIP in the serum. The method of the test was in compliance with the manufacturer’s protocol.

### 16S rDNA sequencing

The whole stomach and duodenum were aseptically taken out and put into sterile EP tubes; liquid nitrogen was quickly put into them and then transferred to a −80˚C refrigerator for testing.

The total DNA was extracted from gastric samples using the FastDNA^®^SPIN Kit for Soil (Omega Bio-tek, Norcross, GA, USA) according to the manufacturer’s instructions. The DNA extract was checked on 1% agarose gel, and DNA concentration and purity were determined with a NanoDrop 2000 UV-vis spectrophotometer (Thermo Scientific, Wilmington, USA). The hypervariable region V3–V4 of the bacterial 16S rDNA gene was amplified with primer pairs 338F (5′-ACTCCTACGGGAGGCAGCAG-3′) and 806R (5′-GGACTACHVGGGTWTCTAAT-3′) by an ABI GeneAmp^®^ 9700 PCR thermocycler (ABI, CA, USA). The PCR amplification of 16S rRNA gene was performed as follows: initial denaturation at 95°C for 3 min, followed by 27 cycles of denaturing at 95°C for 30 s, annealing at 55°C for 30 s, and extension at 72°C for 45 s, and single extension at 72°C for 10 min, and end at 4°C. The PCR mixtures contain 5× TransStart FastPfu buffer of 4 μl, 2.5 mM dNTPs of 2 μl, forward primer (5 μM) of 0.8 μl, reverse primer (5 μM) of 0.8 μl, TransStart FastPfu DNA Polymerase of 0.4 μl, template DNA of 10 ng, and finally ddH2O up to 20 μl. PCR reactions were performed in triplicate. The PCR product was extracted from 2% agarose gel and purified using the AxyPrep DNA Gel Extraction Kit (Axygen Biosciences, Union City, CA, USA) according to the manufacturer’s instructions and quantified using Quantus™ Fluorometer (Promega, USA).

### Illumina MiSeq sequencing

Purified amplicons were pooled in equimolar and paired-end sequenced on an Illumina MiSeq PE300 platform/NovaSeq PE250 platform (Illumina, San Diego, USA) according to the standard protocols by Majorbio Bio-Pharm Technology Co. Ltd. (Shanghai, China). The raw reads were deposited into the NCBI Sequence Read Archive (SRA) database.

### Processing of sequencing data

The raw 16S rDNA sequencing reads were demultiplexed, quality-filtered by fastp version 0.20.0 (Chen et al., 2018a), and merged by FLASH version 1.2.7 (Magoc and Salzberg, 2011) with the following criteria. First, the 300-bp reads were truncated at any site receiving an average quality score of<20 over a 50-bp sliding window, and the truncated reads shorter than 50 bp were discarded; reads containing ambiguous characters were also discarded. Second, only overlapping sequences longer than 10 bp were assembled according to their overlapped sequence. The maximum mismatch ratio of the overlap region is 0.2. Reads that could not be assembled were discarded. Third, samples were distinguished according to the barcode and primers, and the sequence direction was adjusted: exact barcode matching and two nucleotide mismatches in primer matching.

Operational taxonomic units (OTUs) with 97% similarity cutoff (Stackebrandt and Goebel, 1994; Edgar, 2013) were clustered using UPARSE version 7.1, and chimeric sequences were identified and removed. The taxonomy of each OTU representative sequence was analyzed by RDP Classifier version 2.2 (Wang et al., 2007) against the 16S rDNA database (e.g. Silva v138) using confidence threshold of 0.7.

### Statistical analysis

The ELISA results and body weight data were statistically analyzed by GraphPad Prism 8.0, and the data were drawn. The index conforming to the normal distribution adopts the mean ± standard deviation (^*x̄*^±s) for statistical description, and independent sample t-test and one-way analysis of variance (ANOVA) were used for intergroup comparison. The *p*< 0.05 indicated that the difference was statistically significant.

## Result

### Changes of the body weight

There was little difference in the weight of mice in each group before model establishment. The weight of mice in group NC were increased continuously after intragastric administration, whereas those in the other three groups were decreased significantly (*p*< 0.05). From 3 days’ treatment, the body weight of mice in the other three groups began to rise steadily. However, these data in group PUD were slightly lower than that in groups LI10 and ST36. (**Figure 1 D**)

### Efficacy of EA against alcohol-induced PUD

**Figure 2:**
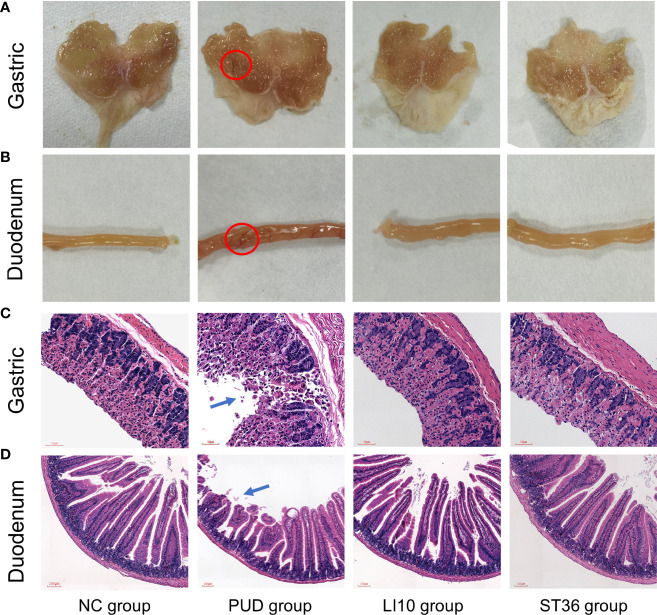
H&E staining and macroscopic observation of gastric and duodenum tissue. **(A)** Observation of gastric tissue with naked eyes. **(B)** Observation of duodenum tissue with naked eyes. **(C)** H&E staining of gastric tissue. **(D)** H&E staining of duodenal tissue.

**Figure 3:**
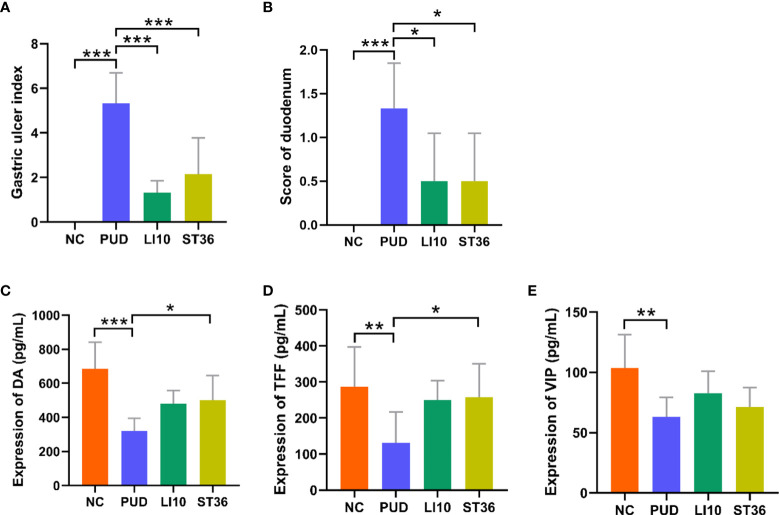
Gastric ulcer index and score of duodenum and expression of DA, TFF, and VIP in serum of mice in each group. **(A)** Gastric ulcer index. **(B)** Score of duodenal injury. **(C)** Expression of dopamine (DA). **(D)** Expression of trefoil factor (TFF). **(E)** Expression of vasoactive intestinal peptide (VIP). *p< 0.05, **p< 0.01, ***p< 0.001.

The mucosa of the stomach and duodenum was observed by naked eye. As shown in **Figures 2A, B**, it can be seen that the tissue morphology of the gastric mucosa in group NC is normal, the mucosal surface is smooth, and the morphology of the duodenum is normal. In group PUD, the gastric mucosa with wired injury and duodenum with congestion and edema were observed. The morphology of the gastric and duodenum in group LI10 and group ST36 tended to be intact. Moreover, there was no obvious injury, bleeding, and ulcer in the mucosa. In addition, the gastric ulcer index and the duodenal score showed that the injury in the model group is significant. After EA treatment, the gastric ulcer index and duodenal injury score of mice in groups LI10 and ST36 were lower than those in the model group (all *p*< 0.01) (**Figures 3A, B**). Furthermore, H&E staining revealed that the gastric mucosa layer in the group PUD mice was damaged obviously. The arrangement of the mucosa cells was disordered, and the local gland cells were broken, fallen off, and sunken to form an ulcer (**Figure 2C**). Compared with the group PUD, the structure of the gastric mucosa and muscle layer in groups LI10 and ST36 basically returned to normal, and the cells arranged orderly. It indicates that EA has a certain repairing effect on the gastric mucosa. Likewise, the duodenal villi were widened and shortened, the crypts became shallow, and some villi were missing in group PUD (**Figure 2D**). The arrangement of duodenal mucosal cells in groups LI10 and ST36 was closer to that in group NC, and the villus morphology basically returned to normal. It shows that EA also has a certain repair effect on duodenal mucosa, too.

### Regulation of EA on DA, TFF and VIP in the serum

The relative expressions of DA (**Figure 3C**), TFF (**Figure 3D**), and VIP (**Figure 3E**) in the serum of all mice were detected by ELISA. Compared with the group NC, the levels of serum DA, TFF, and VIP in group PUD were decreased significantly (all *p*< 0.01). Compared with the group PUD, the levels of serum DA, TFF, and VIP in group LI10 were increased but not significantly different (all *p* > 0.05). However, the levels of DA and TFF in group ST36 increased significantly (all *p*< 0.05). There was no significant difference in relative expression between the groups LI10 and ST36.

### Effect of EA on gastric microbiota in alcohol-induced PUD mice

**Figure 4:**
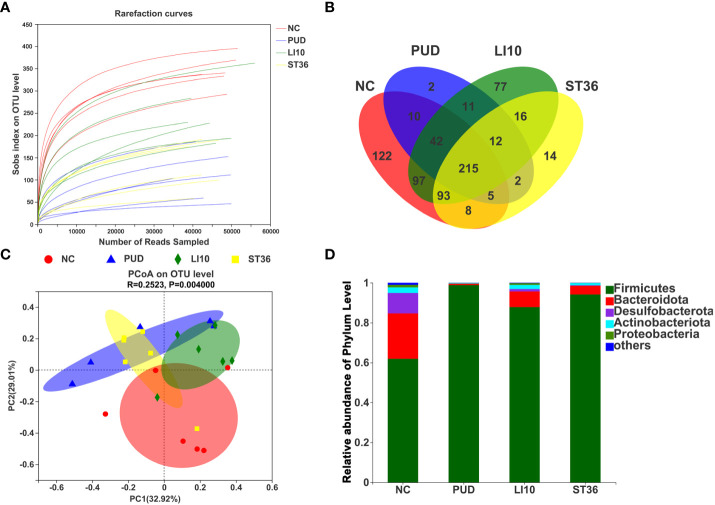
Effect of EA on gastric microbiota in alcohol-induced PUD mice. **(A)** The dilution curve. **(B)** Venn diagram of OTUs for gastric microbiota. **(C)** PCoA analysis of gastric microbiota. **(D)** Composition of gastric microbiota community at phylum level.

A total of 1,819 operational taxonomic units (OTUs) were obtained by high-throughput sequencing of the 16S rDNA gene of the gastric tissue samples using PCR amplification method. As shown in the dilution curve (**Figure 4A**), with sequencing increasing, actual number of species in each group tends to be flat, indicating that this sequencing can reflect the vast majority of microbial diversity information in all mouse gastric tissue samples. The OTU clustering Venn diagram could analyze the common and unique OTUs between different groups. Group NC, PUD, LI10, and ST36 had 215 common OTUs of gastric microbiota and 122, 2, 77, and 16 unique OTUs, respectively (**Figure 4B**).

The results of PCoA analysis (**Figure 4C**) based on Bray–Curtis showed that there were significant differences in the microbiota structure of the gastric tissue among groups NC, PUD, LI10, and ST36, and the relative difference between group PUD and group NC was far. The microbiota profiles of group PUD and groups LI10 and ST36 were separated to some extent. It indicated that the structure of gastric microbiota in PUD mice changed, and EA at LI10 or ST36 could restore those changes.

*Firmicutes*, *Bacteroideta*, *Desulfobacterota*, *Actinobacteriota*, and *Proteobacteria* comprised the main gastric microbiota at the phylum level, but the composition was different in each group (**Figure 4D**). The abundance of *Firmicutes* is the dominant microorganism in mouse gastric tissue; its abundance reached 61.89%, 98.82%, 87.82%, and 94.13% in the NC, PUD, LI10, and ST36 groups, respectively. The abundance of *Bacteroidete*s was second only to *Firmicutes* in mice gastric tissues and reached 22.75%, 0.73%, 7.85%, and 4.39% in NC, PUD, LI10, and ST36 groups, respectively.

**Figure 5:**
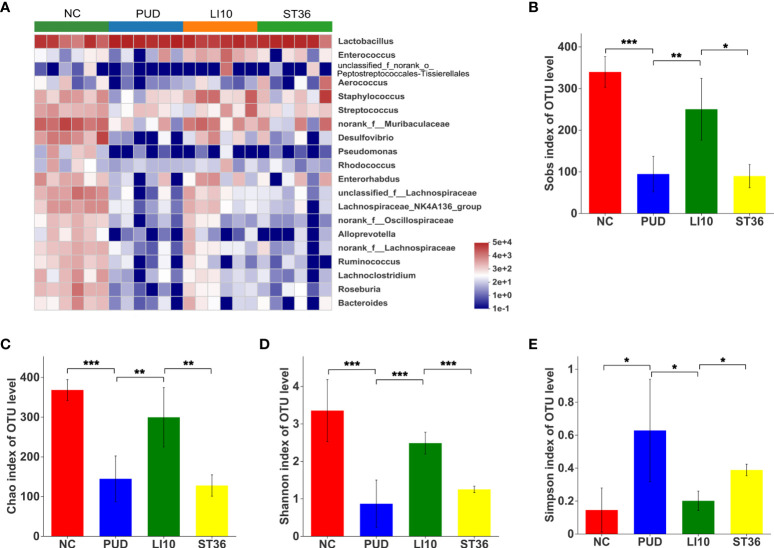
Effect of EA on gastric microbiota in alcohol-induced PUD mice. **(A)** Community heatmap analysis of gastric on genus level. **(B)** Sobs index of gastric microbiota. **(C)** Chao index of gastric microbiota. **(D)** Shannon index of gastric microbiota. **(E)** Simpson index of gastric microbiota. *p< 0.05, **p< 0.01, ***p<0.001.

The top 20 species with relative abundance for gastric microbiota were screened at the genus level, and the cluster analysis was carried out according to the sample relative abundance to draw the heat map (**Figure 5A**). It clearly shows that there is no significant difference between the control group and the rest of the groups. The relative abundances of *Enterorhabdus*, *norank_f_Muribaculaceae*, *Lachnospiraceae_NK4A136_group*, *norank_f_ Lachnospiraceae*, *Lachnoclostridium*, and *Roseburia* in group LI10 are a little more than those in group PUD.

Alpha diversity analysis results show that the Chao, Sobs, and Shannon indexes for the gastric microbiota were significantly decreased, and the Simpson index was increased after model construction (all *p*< 0.05) (**Figures 5B–E**), indicating a lower microbial community richness and diversity. Compared to group PUD, the Chao, Sobs, and Shannon indexes of group LI10 were significantly increased (all *p*< 0.05), while those in group ST36 were no significant difference (all *p* > 0.05); the Simpson index of group LI10 was decreased, while that in group ST36 had no significant difference (*p*< 0.05, *p* > 0.05).

### Effect of EA on duodenal microbiota in alcohol-induced PUD mice

**Figure 6:**
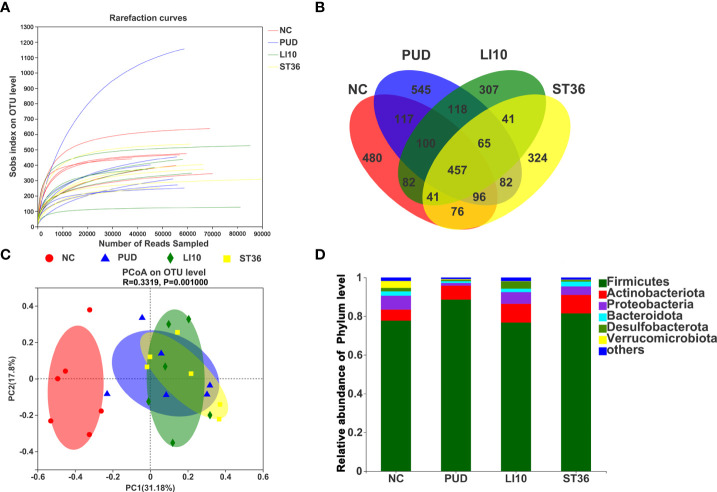
Effect of EA on duodenal microbiota in alcohol-induced PUD mice. **(A)** The dilution curve of duodenal microbiota. **(B)** Venn diagram of OTUs for duodenal microbiota. **(C)** PCoA analysis of duodenal microbiota. **(D)** Composition of duodenal microbiota community at phylum level.

A total of 5,422 OTUs were obtained by high-throughput sequencing of the 16S rDNA gene of the duodenum tissue samples using the same method. As shown in the dilution curve (**Figure 6A**), with sequencing increasing, actual number of species in each group tends to be flat, indicating that this sequencing can reflect the vast majority of microbial diversity information in all mouse duodenum tissue samples. The OTU clustering Venn diagram could analyze the common and unique OTUs between different groups. Groups NC, PUD, LI10, and ST36 had 457 common OTUs of duodenal microbiota and 480, 545, 307, and 324 unique OTUs, respectively (**Figure 6B**).

The results of the principal coordinates analysis (PCoA) (**Figure 6C**) based on Bray–Curtis showed that there were significant differences in the microbiota structure of the duodenum tissue among groups NC and PUD. However, there was little difference between groups LI10 and ST36 and PUD. This indicated that EA had no effect on the duodenal microbiota.

*Firmicutes*, *Actinobacteriota*, *Desulfobacterota*, *Proteobacteria*, *Bacteroideta*, and *Verrucomicrobiota* comprised the main duodenal microbiota at the phylum level (**Figure 6D**). *Firmicutes* is still the microbiota with the highest relative abundance in each group, reaching 77.73%, 88.58%, 76.76%, and 81.49% in the NC, PUD, LI10, and ST36 groups, respectively. The abundance of *Bacteroidetes* is 2.20%, 0.91%, 1.75%, and 2.50% in NC, PUD, LI10, and ST36 groups, respectively.

**Figure 7:**
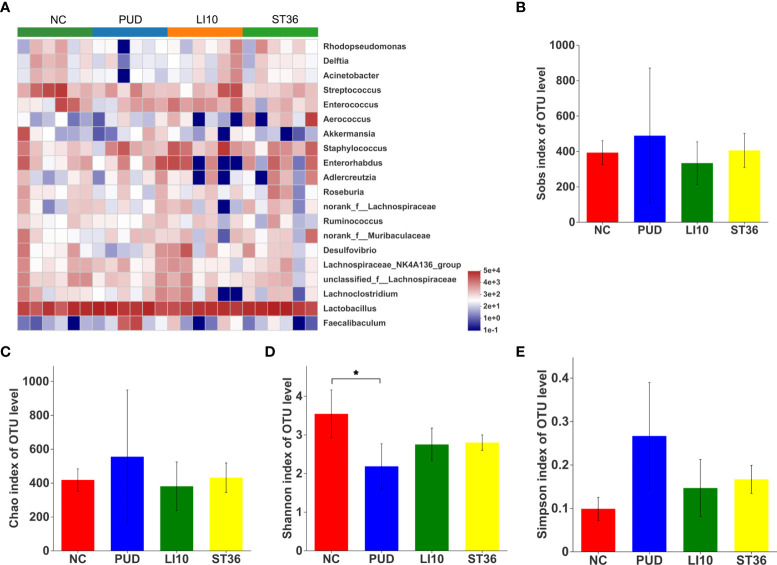
Effect of EA on duodenal microbiota in alcohol-induced PUD mice. **(A)** Community heatmap analysis of duodenum on genus level. **(B)** Sobs index of duodenal microbiota. **(C)** Chao index of duodenal microbiota. **(D)** Shannon index of duodenal microbiota. **(E)** Simpson index of duodenal microbiota. *p< 0.05.

The top 20 species with relative abundance for duodenal microbiota were screened at the genus level, and the cluster analysis was carried out according to the sample relative abundance to draw the heat map (**Figure 7A**). It can be seen that the duodenal microbial community structure of the four groups is not significantly different.

Alpha diversity analysis results show that the Shannon index was decreased after model construction (*p* > 0.05, *p* > 0.05, *p*< 0.05, and *p*< 0.05) (**Figure 7D**), indicating a lower microbial community diversity. However, the Chao, Sobs, and Simpson indexes shows no difference (all *p* > 0.05) (**Figures 7B–E**). These results suggested that there was no significant change in the alpha diversity of duodenal microbial after EA.

## Discussion

PUD is still a common disease encountered worldwide, with an incidence rate of 0.1%–0.3% every year. PUD affects approximately 5%–10% of the global population, and its presentation varies according to age, gender, and geographical location (Lanas and Chan, 2017). *Helicobacter pylori* infection is a high-risk factor for the development of PUD. In addition, long-term or excessive drinking can cause gastric mucosal damage or even bleeding, resulting in PUD (Huang et al., 2021). Ethanol is one of the most irritating exogenous substances that can damage the gastric mucosa. It can quickly penetrate the gastric mucosal layer in a short time, causing damage to the membrane and leading to tissue erosion (Sidahmed et al., 2013), endogenous mucus rupture, and increased gastric acid secretion. These effects result in the development of hemorrhagic lesions and gastric mucosal injury (Rajasekaran et al., 2012). An ethanol-induced peptic ulcer disease murine model has been widely used in the study of the mechanism of anti-ulcer drugs (Zhang et al., 2019).

The gastrointestinal tract is a complex microbial ecosystem, containing trillions of different microorganisms (bacteria, fungi, archaea, and viruses). The gastrointestinal microbiota plays an important role in the occurrence and development of PUD (Schulz et al., 2016; Rooks and Garrett, 2016). Due to the presence of gastric acid, the number and species of the microbiota present in the stomach are different from those noted in other intestinal segments (Bik et al., 2006; De Witte et al., 2016). Therefore, it is necessary to explore the gastric microbiota in the study of PUD. ST36 is one of the most commonly used acupoints in the clinical treatment of PUD (Tian et al., 2017). Previous studies (He et al., 2019) have shown that moxibustion at ST36 and Liangmen (ST21) can increase the abundance of *Alphaproteobacteria* and actinomycetes in the feces, improve the microbial diversity of feces, and ameliorate the damage of gastric mucosa. Similarly, another study (Wang et al., 2020) has shown that acupuncture at Baihui (GV20), Zhongwan (CV12), and ST36 can increase the abundance of *Bacteroidetes* in the feces and reduce the abundance of *Firmicutes* and *Proteobacteria*. It should be noted that the bacterial community in feces does not reflect the overall intestinal microbiota of the entire gastrointestinal tract. The microbiota in the stomach and duodenum is closely related to the PUD and may be neglected. In the present study, the determination of the ulcer index and the pathological results indicated that bleeding, injury, and ulcer changes occurred in the gastric mucosal tissues, and congestion, edema, and inflammation occurred in the duodenal tissues of the PUD animals. By contrast, gastroduodenal injury and inflammation were improved in groups LI10 and ST36. The data indicated that EA at ST36 could improve gastric and duodenal mucosal injury, which is consistent with the findings reported in previous studies (Zhang et al., 2018; Wang et al., 2021). The treatment effect of EA at LI10 on the injury caused in the gastrointestinal mucosa is noteworthy, and this finding has not been reported in previous studies. LI10 and ST36 both belong to the Yangming Meridian. Their positions are symmetrical up and down. Recent studies (Liu et al., 2022) have shown that EA at LI10 or ST36 can exert anti-inflammatory effects *via* the same pathway. Exploring the effect of EA at these two acupoints on the gastroduodenal microbiota will be helpful to further reveal its mechanism. Our research suggested that the two acupoints have the same effect on the repair of the gastrointestinal mucosa, while acupuncture at LI10 caused a more significant improvement in the richness and diversity of the gastric microbiota. The loss of microbial diversity is regarded as one of the indicators of the presence of intestinal diseases (Mosca et al., 2016). *Firmicutes* and *Bacteroidetes* are the main phyla of the mouse gastric microbiota. *Firmicutes* plays an important role in energy absorption, which is closely related to the digestion and dietary absorption (Wang et al., 2015). *Bacteroidetes* are the main defense of the gastrointestinal immune system (Ley et al., 2006). Previous studies have shown that *H. pylori*-infected mice have increased *Firmicutes* and decreased *Bacteroidetes* contents in the stomach, which can further aggravate the damage caused to the gastric mucosa (Lofgren et al., 2011). In addition, the increase in the proportion of *Firmicutes*/*Bacteroidetes* may alter the intestinal environment so as to improve immune function and promote intestinal health (Stojanov et al., 2020). Furthermore, at the genus level, the abundance of *Lactobacillus* was decreased following treatment with EA. Although *Lactobacillus* is a beneficial bacterium to healthy subjects, it may also become conditional pathogens under different circumstances, which can have a serious impact on the host with chaotic intestinal microbiota (Shang et al., 2018; Keikha and Karbalaei, 2021). It has been reported that there are more than 200 cases of *Lactobacillus*-related infections that are present in patients with ulcerative colitis, short bowel syndrome, and cancer (Cannon et al., 2005). The present study indicated that the changes in the relative abundances of *Firmicutes* and *Bacteroidetes* in the gastric microbiota may be responsible for the ability of EA to ameliorate PUD. Furthermore, EA at LI10 improved the diversity and richness of gastric microbiota.

In addition, the role of DA, TFF, and VIP in the occurrence and development of PUD cannot be ignored. Some studies have shown that regulating DA can ameliorate gastric ulcer (Leng et al., 2019). TFF is a small molecular polypeptide secreted by gastrointestinal mucus cells. It has been shown to have a protective effect on the gastrointestinal mucosa and promote mucosal healing (Galura et al., 2019; Yang et al., 2022). VIP can maintain the steady state of the gastric mucosa and induce the protective effect of the gastric mucosa under certain conditions (Gyires, 2004). Furthermore, accumulating evidence has shown that the gut microbiota interacts with the enteric and central nervous systems (ENS and CNS, respectively) *via* communication along the “gut–brain axis” (Fung et al., 2017). It has also been shown that bacteria have the ability to produce a series of neurotransmitters (Strandwitz, 2018). For example, in the presence of dopamine, the growth rates of *Escherichia*, *Staphylococcus*, and *Bacillus* were increased (Tsavkelova et al., 2000). Although it has not been confirmed that the microbiota regulates dopamine *in vivo*, accumulating evidence suggests that it may play a role in host biosynthesis/catabolism. VIP widely acts as a neurotransmitter/neuromodulator in the central and peripheral nervous systems. VIP maintains the normal barrier function of the gastrointestinal tract by promoting the differentiation, proliferation, and cell adhesion of intestinal epithelial cells (IEC) (Wu et al., 2015). The VIP neuropeptide plays an important role in intestinal homeostasis in mice. Lack of VIP is related to changes in the structure of the microbiota, biodiversity, and weight loss, which provides evidence for its impact the regulation of the intestinal bacterial structure (Bains et al., 2019). The changes in DA and VIP levels and gastric microbiota may be associated with the microbiota–gut–brain axis (Cryan et al., 2019). Previous studies have shown that drinking alcohol may reduce TFF and damage gastrointestinal mucosal barrier, thus affecting gastrointestinal microbiota (Shao et al., 2015). Moreover, EA at ST36 can increase the levels of DA and TFF in the serum and reduce the level of VIP (Li et al., 2006) (Li et al., 2016). Our results show that EA at ST36 can also increase DA and TFF, but it has little effect on VIP; the influence of EA at LI10 on DA and TFF is slightly weaker than that of EA at ST36. It can be speculated that EA alleviating PUD is related with the levels of DA and TFF and the gastric microbiota.

This study also has limitations. Due to the lack of clear control conditions (sham acupuncture), the influence of the operation itself cannot be ruled out. This may lead to an incomplete conclusion. Another potential limitation is the lack of a clear mechanism explanation to clarify the basic physiological principles of EA. In future studies, the clear control conditions (sham acupuncture) and more in-depth mechanism explanation should be considered. If the gastrointestinal microbiota can be artificially regulated by EA, EA will become a great complementary and alternative therapy for many diseases.

## Conclusion

Our study suggested that EA could promote repair of the gastroduodenal mucosa, improve the levels of DA and TFF, and impact the abundance of *Firmicutes* and *Bacteroidetes*.

## Data availability statement

The datasets presented in this study can be found in online repositories. The names of the repository/repositories and accession number(s) can be found below: https://www.ncbi.nlm.nih.gov/,SRA:SRP375580.

## Ethics statement

This study was reviewed and approved by Animal Protection and Ethics Committee of Xiamen University.

## Author contributions

ZY and XL gave the direction of the paper’s conception. XL, FH, and XT performed the experiments. XL, JG, and YQ performed the statistical analysis. XL wrote the paper, and YQ proofread the manuscript. ZY and XM provided the intellectual support and modified language. YW participated in the revision of manuscripts. All authors contributed to the article and approved the submitted version.

## Funding

This project was supported by the National Natural Science Foundation of China (NO.81973934).

## Acknowledgments

We acknowledge the help from the Experimental Animal Center of Xiamen University.

## Conflict of interest

The authors declare that the research was conducted in the absence of any commercial or financial relationships that could be construed as a potential conflict of interest.

## Publisher’s note

All claims expressed in this article are solely those of the authors and do not necessarily represent those of their affiliated organizations, or those of the publisher, the editors and the reviewers. Any product that may be evaluated in this article, or claim that may be made by its manufacturer, is not guaranteed or endorsed by the publisher.
